# Structure-Function Relationship of XCL1 Used for *in vivo* Targeting of Antigen Into XCR1^+^ Dendritic Cells

**DOI:** 10.3389/fimmu.2018.02806

**Published:** 2018-12-13

**Authors:** Arthur L. Kroczek, Evelyn Hartung, Stephanie Gurka, Martina Becker, Nele Reeg, Hans W. Mages, Sebastian Voigt, Christian Freund, Richard A. Kroczek

**Affiliations:** ^1^Molecular Immunology, Robert Koch-Institute, Berlin, Germany; ^2^Protein Biochemistry, Institute for Biochemistry, Free University of Berlin, Berlin, Germany; ^3^Institute of Biochemistry, Charité University Medicine Berlin, Berlin, Germany; ^4^Virology, Robert Koch-Institute, Berlin, Germany

**Keywords:** dendritic cells, XCR1, XCL1, cross-presentation, antigen targeting

## Abstract

XCL1 is the ligand for XCR1, a chemokine receptor uniquely expressed on cross-presenting dendritic cells (DC) in mouse and man. We are interested in establishing therapeutic vaccines based on XCL1-mediated targeting of peptides or proteins into these DC. Therefore, we have functionally analyzed various XCL1 domains in highly relevant settings *in vitro* and *in vivo*. Murine XCL1 fused to ovalbumin (XCL1-OVA) was compared to an N-terminal deletion variant lacking the first seven N-terminal amino acids and to several C-terminal (deletion) variants. Binding studies with primary XCR1^+^ DC revealed that the N-terminal region stabilizes the binding of XCL1 to its receptor, as is known for other chemokines. Deviating from the established paradigm for chemokines, the N-terminus does not contain critical elements for inducing chemotaxis. On the contrary, this region appears to limit the chemotactic action of XCL1 at higher concentrations. A participation of the XCL1 C-terminus in receptor binding or chemotaxis could be excluded in a series of experiments. Binding studies with apoptotic and necrotic XCR1-negative cells suggested a second function for XCL1: marking of stressed cells for uptake into cross-presenting DC. *In vivo* studies using CD8^+^ T cell proliferation and cytotoxicity as readouts confirmed the critical role of the N-terminus for antigen targeting, and excluded any involvement of the C-terminus in the uptake, processing, and presentation of the fused OVA antigen. Together, these studies provide basic data on the function of the various XCL1 domains as well as relevant information on XCL1 as an antigen carrier in therapeutic vaccines.

## Introduction

Murine XCL1 is a chemokine of 93 amino acids, and has been originally identified as lymphotactin by Kelner et al. (1), while human XCL1 was found by us (2), and by Yoshida et al. (3). Mature murine XCL1 exhibits a high degree of homology to human XCL1 (also 93 aa), with 61% identity and 84% similarity, and both homologs have an identical structure [for alignment please refer to Geyer et al. (4)]. The XCL1/XCR1 chemokine ligand-receptor pair exhibits some special structural and functional features.

XCL1 is secreted by activated NK cells, activated Th1-polarized CD4^+^ T cells, and activated CD8^+^ T cells, and co-secreted with IFN-γ, MIP-1α, MIP-1β, and RANTES and is thus part of the Th1 immune defense (5, 6). XCR1 is the only receptor for XCL1, and XCL1 is the only ligand of this receptor (7, 8). Thus, this ligand/receptor pair is monogamous, a rare feature in the world of around 50 chemokines.

The receptor XCR1 is exclusively expressed on a subset of dendritic cells (DC), the “cross-presenting” DC (and not elsewhere in the body), in the mouse, the rat, and the human (4, 9–13). This narrow expression spectrum is another unusual feature of the XCL1/XCR1 axis. XCR1^+^ DC, earlier designated as CD8^+^ DC in the mouse, were demonstrated to be particularly efficient in the uptake of cells stressed by (intracellular) infection (14–16). Moreover, CD8^+^ DC have consistently been shown to excel in antigen “cross-presentation,” in which exogenous antigen is not presented in the context of MHC class II to CD4^+^ T cells, but instead shunted into the MHC class I pathway of antigen presentation to CD8^+^ T cells (17–19). Given the secretion profile of XCL1, XCR1^+^ DC can be regarded as a DC population closely cooperating with NK cells, Th1-polarized CD4^+^ T cells, and CD8^+^ T cells in the surveillance of stressed/ transformed cells for “danger” (16). The cross-presenting XCR1^+^ DC are now also commonly referred to as cDC1.

In the past, we have employed this highly specific expression of XCR1 to target antigens into cross-presenting DC *in vivo*. In these experiments, ovalbumin (OVA), recombinantly fused to the C-terminal portion of murine XCL1 (“XCL1-OVA”), was highly efficient in inducing antigen-specific CD8^+^ T cell cytotoxicity, when compared to untargeted OVA (20). These experiments demonstrated that XCL1 can be employed as a carrier for therapeutic vaccines intended to elicit potent antigen-specific T cell cytotoxicity *in vivo*.

Because of this therapeutic potential, we are interested in the structure-function relationship of various domains of XCL1. Like classical chemokines, XCL1 has a free N-terminus of around 10 amino acids (aa), which is followed by a structured core domain of around 60 aa containing a three-stranded antiparallel beta-sheet and a C-terminal alpha-helix (classical “chemokine fold”). The C-terminal portion of XCL1 of around 20 aa is, typical for chemokines, again unstructured (21). The C-terminus is highly conserved between mouse, rat, and human XCL1, and of unknown function.

XCL1 is the only chemokine with one disulphide bridge, while all other chemokines stabilize their tertiary structure by two disulphide bridges. Kuloglu et al. (22) have demonstrated *in vitro* that due to the lack of this second disulphide bridge, XCL1 can assume at some more extreme conditions (45°C, no salt) an alternative conformation (which is exceptional in the chemokine world), which could imply a second function. This unusual feature raised the question whether the various domains of XCL1 functionally differ from classical chemokines or whether XCL1 has more than one function.

To fully understand the usefulness of XCL1 as a vector system for protein vaccines, we set out to systematically test the contribution of XCL1-domains on receptor binding, its chemotactic function, and on antigen processing and presentation to CD8^+^ T cells *in vivo*. To this end, N-terminal and C-terminal deletion variants of XCL1-OVA (which we have previously used for antigen targeting *in vivo* [Hartung et al. (20), see above] were generated. Further, we also replaced the entire C-terminal domain of XCL1 with the C-terminal domain of viral XCL1 (vXCL1), a rat cytomegalovirus-encoded XCL1 homolog, which we have recently identified and characterized (4). vXCL1, which can be assumed to interfere with the immune defense, has a fully intact chemotactic activity on cross-presenting DC and mainly differs in its C-terminal portion from its rat homolog. We thus utilized the viral C-terminus in order to determine whether this domain in some ways contributes to the function of XCL1.

## Materials and Methods

### Cloning, Expression, and Purification of XCL1-OVA and Its Structural Variants

The DNA fragments coding for the various XCL1-OVA constructs with a C-terminal Strep-tag (IBA, Germany) were cloned into the drosophila expression vector pRmHa-3 (23) by standard procedures. XCL1-OVA encoding plasmids were electroporated together with the plasmid phshs.PURO into drosophila Schneider SL-3 cells (24) using a Bio-Rad Gene Pulser (450 V and 500 mF). The phshs.PURO plasmid (kindly provided by M. McKeown, Salk Institute) allows selection of positive transfectants by puromycin. Clones from limiting dilution cultures of transfected SL-3 cells were induced with 1 mM CuSO_4_ and analyzed for high protein production using either XCL1- or Strep-tag-specific ELISA. Positive clones were expanded in Insect-XPRESS medium (Lonza) on a shaker platform (100 rpm) in normal air at 27°C. XCL1-OVA proteins were purified from supernatants using Strep-Trap HP columns from GE Healthcare according to the manufacturer's instructions. Protein concentration was determined by measuring OD_280_ using a Nanodrop ND-1000 (Thermo Scientific). LPS content in all protein samples was <0.5 EU/mg protein.

### Mice

C57BL/6 mice (8–12 week old) were used for experiments and cell isolation, unless indicated otherwise. B6.XCR1-lacZ (The Jackson Laboratory) are XCR1-deficient mice in which the XCR1 gene has been replaced by the β-Gal reporter gene; these mice were fully backcrossed (>10x) onto the C57BL/6 background. OT-I TCR-transgenic mice were crossed onto the B6.PL background to allow identification of CD8^+^ T cells using the CD90.1 marker. All mice were bred under specific pathogen-free conditions in the animal facility of the Federal Institute for Risk Assessment (Berlin, Germany). All animal experiments were performed according to state guidelines and approved by the local animal welfare committee.

### Cell Isolation

Splenocytes were obtained by mashing spleens through 70 μm cell sieves into PBS, followed by erythrocyte lysis with ACK Buffer (155 mM NH_4_Cl, 10 mM KHCO_3_, 0.1 mM EDTA).

### Chemotaxis

To obtain sufficient DC for chemotaxis assays, C57BL/6 mice were injected s.c. at 2 sites, each site with 1.5 × 10^6^ B16 cells secreting Flt3 ligand (25) for 9 days. DC were then enriched by cutting spleens into small pieces followed by digestion with Collagenase D (500 μg/ml) and DNase I (20 μg/ml, both Roche) for 25 min at 37°C in RPMI 1640 containing 2% FCS (low endotoxin, Biochrom); EDTA (10 mM) was added for additional 5 min and cells were filtered through a 70 μm nylon sieve (BD Falcon). DC were further enriched by centrifugation over a 1.073 g/ml density gradient (NycoPrep, Axis-Shield). For chemotaxis assays, 1 × 10^6^ DC (purity ~70%) were suspended in 100 μl chemotaxis medium (RPMI 1,640, 1% BSA, 50 mM β-ME, 100 mg/ml penicillin/streptomycin) and placed into the upper chamber of a 24-well Transwell system (6.5 mm diameter, 5-μm pore polycarbonate membrane; Corning Costar). The lower chamber was filled with chemotaxis medium to which recombinant XCL1-OVA or the XCL1-variants were added. After incubation in 5% CO_2_ for 2 h at 37°C, the number of migrated DC was determined by counting cells in the lower chamber using a flow cytometer. DC were identified by staining for XCR1, CD8, CD11c, and MHC II after gating out cells expressing B220 and CD3. The percentage of migrated cells was calculated by dividing the number of cells in the lower chamber by the number of input cells (number migrated cells/number input cells × 100).

### *In vivo* Proliferation of OT-I T Cells

Recipient C57BL/6 mice were adoptively transferred with splenocytes containing 1 × 10^6^ OT-I (CD8^+^) resting T cells (negative for CD25, CD69, and ICOS). For proliferation analysis, OT-I cells were labeled with 5 μM CFSE (Invitrogen) before transfer and analyzed 48 h after immunization using the CFSE dilution assay. Detection of OT–I T cells after adoptive transfer was with mAb to CD8 and CD90.1 after gating out CD4^+^ T cells and B220^+^ cells.

### *In vivo* Cytotoxicity Assay

Animals were immunized with the indicated amounts of either XCL1-OVA or the respective variants together with 3 μg of LPS (Sigma), which was mixed with the protein variants before injection i.v. 6 days later, 10 × 10^6^ syngeneic splenocytes were pulsed with SIINFEKL peptide (GenScript) and labeled with 10 μM CFSE (CFSE^high^) *in vitro*, while 10 × 10^6^ splenocytes were left unpulsed and labeled with 1 μM CFSE (CFSE^low^). Both preparations were injected together i.v. into immunized and control animals, and the CFSE signal was determined by flow cytometry 18 h later. Specific lysis was calculated using the following formula: specific lysis (%) = 100–([CFSE^high^ immunized/CFSE^low^ immunized]/[CFSE^high^ control/CFSE^low^ control]) × 100.

### Antibodies and Staining Reagents

Hybridomas producing mAb recognizing CD4 (clone YTS 191.1), CD8 (53–6.72), CD11c (N418), CD44 (IM7.8.1), CD45R (B220 clone RA3-6B2), CD62L (MEL-14), Ly6G/C (RB6-8C5), and MHC class II (M5/114.15.2) were obtained from ATCC, CD90.1 (OX-7) from ECACC. Mab to CD69 (H1.2F3) was from Biolegend, mAb PD-1 (J43) from eBioscience. Anti-CD3 (KT3) was generously provided by H. Savelkoul, anti-CD25 (2E4) by E. Shevach. Generation of anti-XCR1 mAb MARX10 (13) and anti-ICOS mAb [MIC-280 (26)] has been described before. Mab MTAC-311 detects the C-terminal part of murine XCL1 (unpublished antibody and data). Generation of XCL1-StrepTag is described in Hartung et al. (20). The non-agonistic mAb MARX10 (mouse IgG2b, in the recombinant version IgG1) does not block the binding of XCL1 to XCR1. OT–I T cells were identified with mAbs to Vα2-TCR (B20.1, eBioscience) and Vβ5-TCR (MR9-4, BD Biosciences). StrepMAB Immo conjugated to Oyster 645 was from IBA Lifesciences.

### Flow Cytometry

Antibodies were titrated for optimal signal-to-noise ratio. To block unspecific binding to Fc-receptors, cells were pre-incubated with 100 μg/ml 2.4G2 mAb. Standard staining with mAb was in PBS, 0.25% BSA, 0.1% NaN_3_ for 25 min on ice. For exclusion of dead cells 4′,6-diamidino-2-phenylindole (DAPI) was added 5 min before measurement. Doublets and autofluorescent cells were excluded from the analysis. Data were acquired on a LSRII cytometer (BD Biosciences), and analyzed using FlowJo (Tree Star Inc.). DC were defined as CD11c^+^ MHC-II^+^ Lin^−^ cells.

### Stressed Cells Assay

P3X63Ag8.653 myeloma cells (ATCC) were cultured at a density of 2 × 10^6^ cell/ml in complete RMPI1640 medium. Some cells were exposed to heat shock (52°C for 15 min) and thereafter cultured at a density of 2 × 10^6^ cells/ml in a 6-well plate overnight at 37°C and 5% CO_2_. Then, 0.5 × 10^6^ cells were transferred into 24-well culture plate wells and 1 μg or 2 μg of wt XCL1-OVA or one of its variants were added for the last hour of culture. The cells were washed and stained with mAb StrepMAB Immo conjugated to Oyster 645 (to detect bound XCL1-OVA) and Annexin V-Cy5 (BD Pharmingen) in a binding buffer (10 mM Hepes, 140 mM NaCl, 2,5 mM CaCl2, 1% NaN3). After a further washing step, cells taken up in binding buffer and were analyzed by flow cytometry, DAPI was added just before analysis.

## Results

### Generation of XCL1-OVA and the Structural Variants Del-N7, Del-C7, Del-C17, and vCterm

**Figure 1:**
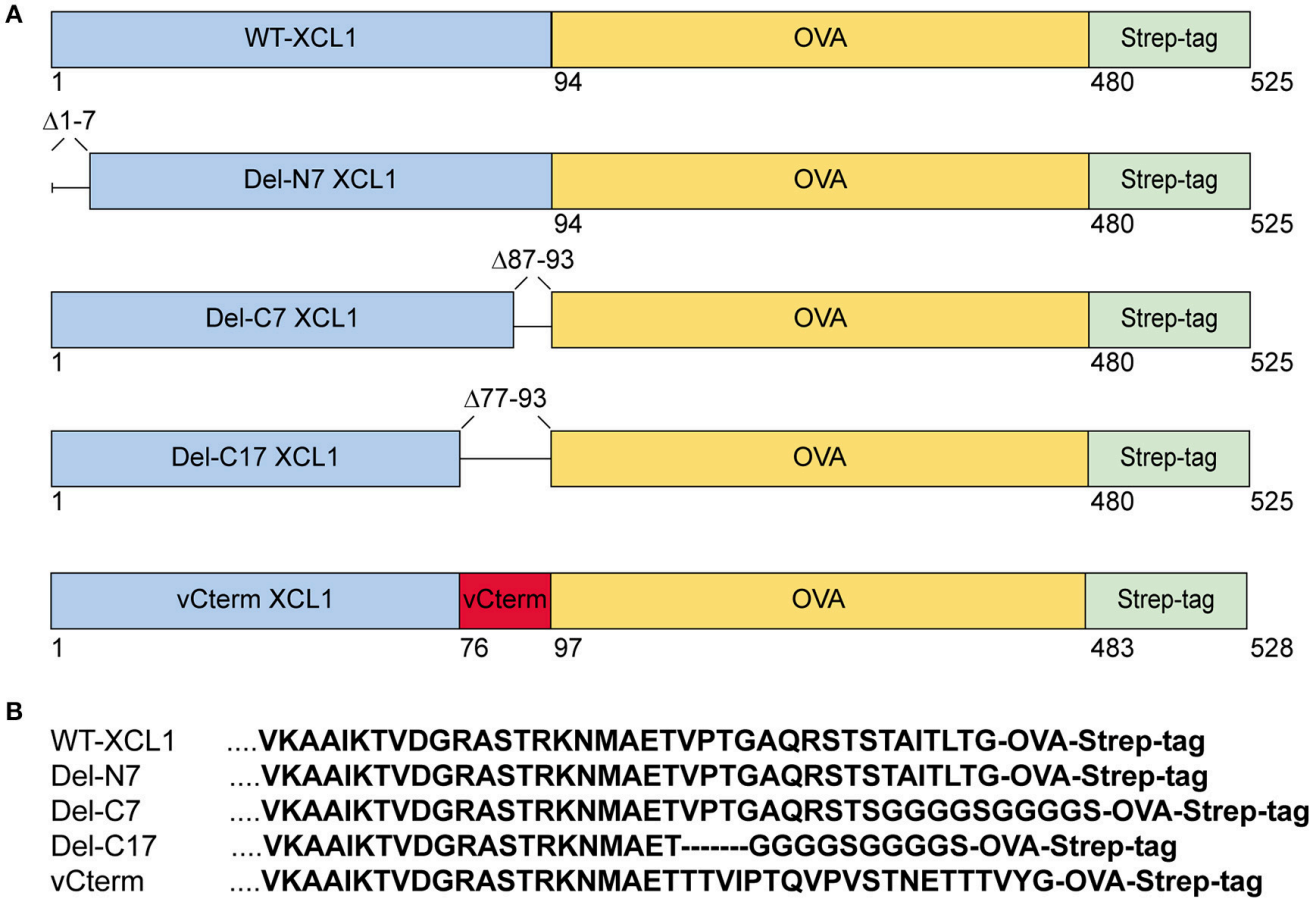
XCL1-OVA and its variants Del-N7, Del-C7, Del-C17, and vCterm. **(A)** Schematic representation of XCL1-OVA and its variants. Del-N7 lacks the first seven amino acids (aa), in Del-C7 and Del-C17, the final 7 and 17 C-terminal aa were deleted, respectively. In vCterm, the 17 C-terminal aa were replaced by the 20 C-terminal aa of murid herpesvirus 8-encoded XCL1. **(B)** C-terminal aa sequences of XCL1 in the respective protein variants.

Various formats of XCL1 recombinantly fused to OVA were generated in order to test for the impact of the various domains (i) on the function of murine XCL1, (ii) on the ability of XCL1 to target the antigen OVA into XCR1^+^ DC *in vivo*, and (iii) on the capacity of XCR1^+^ DC to process and cross-present the attached antigen. The design, production, and *in vivo* targeting capacity and specificity of XCL1-OVA, used here as the standard for comparison, has been described earlier (20). Del-N7 XCL1 is a variant lacking the first seven N-terminal amino acids (aa) of XCL1, but is otherwise identical to XCL1-OVA (Figure 1A). Del-C7 XCL1 lacks the C-terminal 7 aa, Del-C17 the C-terminal 17 aa of XCL1; in both deletion variants a glycine-serine linker (GGGGS) was introduced C-terminally in order to (partially) compensate for any size/positional effects (Figure 1B). vCterm XCL1 is a variant in which the 17 C-terminal aa of murine XCL1 were replaced by the 20 C-terminal aa of a rat cytomegalovirus-encoded XCL1 homolog (4). All constructs contained a C-terminal Strep-tag to allow detection of the bound protein variants to XCR1. The various constructs are represented schematically in Figure 1A. For clarity, the C-terminal sequences of the XCL1-variants are also shown (Figure 1B). The constructs were used to express the protein in Schneider cells.

### The N-Terminus of XCL1 Is Critical for Binding to XCR1

**Figure 2:**
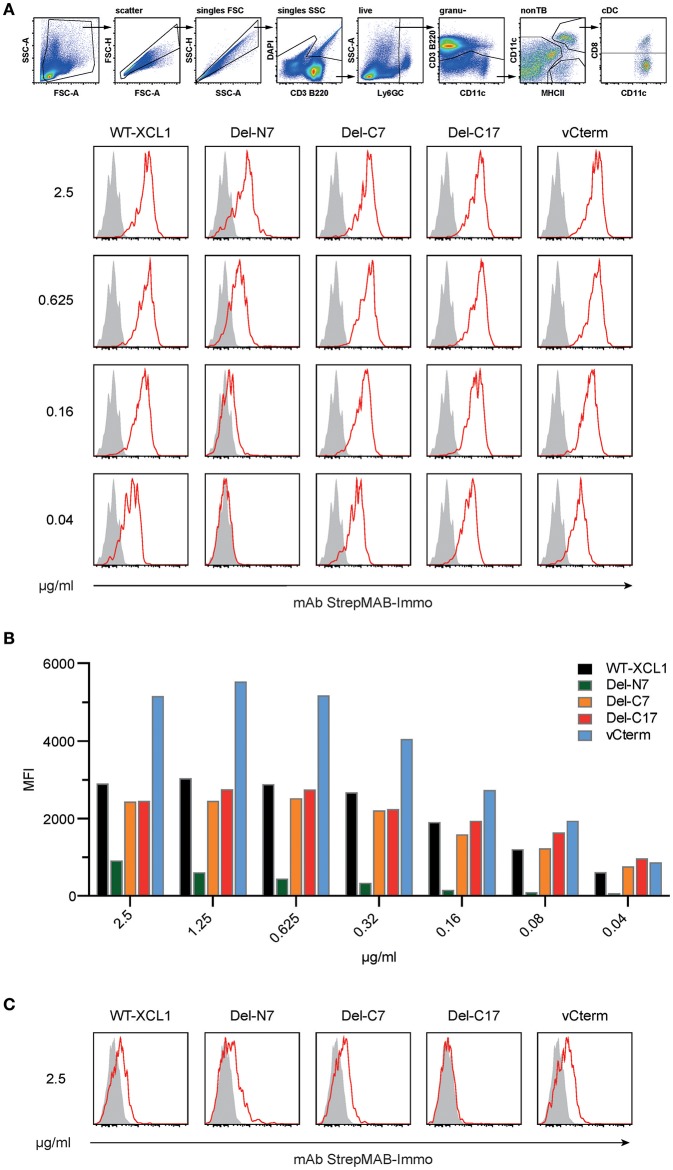
Binding of XCL1 and the structural variants Del-N7, Del-C7, Del-C17, and vCterm to XCR1 expressed on primary dendritic cells. Splenocytes were incubated with carefully titrated (0.04, 0.08, 0.16, 0.315, 0.625, 1.25, and 2.5 μg/ml) concentrations of all XCL1-OVA protein variants (all concentrations are given based on the XCL1-component of the constructs) for 25 min on ice, washed, and the bound protein was detected on Ly6G/C^−^ CD3^−^ B220^−^ CD8^+^ CD11c^+^ MHC-II^+^ cells using an anti-Strep-tag mAb StrepMAB-Immo and flow cytometry (red histograms). Background staining, without pre-incubation, using StrepMAB-Immo is shown in gray. **(A)** Signals obtained with XCL1-variants at 0.04, 0.16, 0.625, and 2.5 μg/ml. **(B)** Graphical representation of the mean fluorescence intensity (MFI) obtained in flow cytometry with all protein concentrations of the XCL1-OVA variants used. **(C)** Signals (shown for 0.625 and 2.5/μg/ml) obtained on CD8^+^ DC lacking XCR1. Data are representative of 3 independent experiments.

The binding of XCL1-OVA and its structural variants to its receptor XCR1 was determined by incubating splenocytes with carefully titrated concentrations of each reagent for 25 min on ice, followed by washing. The bound protein variants on XCR1^+^ DC were then detected using an anti-Strep-tag mAb and flow cytometry. Some of the results (incubation of the cells at 2.5, 0.625, 0.16, and 0.04 μg/ml) are represented in histograms in Figure 2A. Figure 2B summarizes experimental data points obtained with all concentrations of the respective protein variants. Small concentrations of XCL1-OVA (0.04 μg/ml) sufficed to achieve substantial binding, and saturation was achieved at around 0.5 μg/ml. The Del-C7 and Del-C17 structural variants showed a binding pattern comparable to wildtype XCL1-OVA (Figures 2A,B). In contrast, the Del-N7 variant only bound at high concentrations of protein. At 2.5 μg/ml, the binding efficiency of Del-N7 was comparable to binding of XCL1-OVA at around 0.05 μg/ml, and thus was diminished around 50-fold. Incubation with vCterm XCL1 resulted in clearly stronger binding signals, when compared to XCL1-OVA (Figures 2A,B).

To control for unspecific signals, the binding experiments were repeated with cells from XCR1-deficient (XCR1^−/−^) mice on the same C57BL/6 background. As can be seen from Figure 2C, all variants exhibited a similar XCR1-unspecific binding (only at this high concentration, data not shown), with the exception of the Del-C17 variant.

These experiments determined that the first seven N-terminal aa of XCL1 have a major influence on the binding of XCL1 to its receptor, which can be partially compensated at high protein concentrations by other structural elements of XCL1. At the same time, the experiments excluded any significant contribution of the C-terminal 17 aa to the binding of XCL1 to XCR1. Binding studies with XCR1^−/−^ dendritic cells further demonstrated that all binding of XCL1 to the DC is mediated by XCR1; the unspecific signals obtained at higher protein concentrations (2.5 μg) are apparently mediated by the C-terminal portion of XCL1 and are most likely of no major relevance *in vivo*.

### Effects of the Structural Variants on Chemotaxis

**Figure 3:**
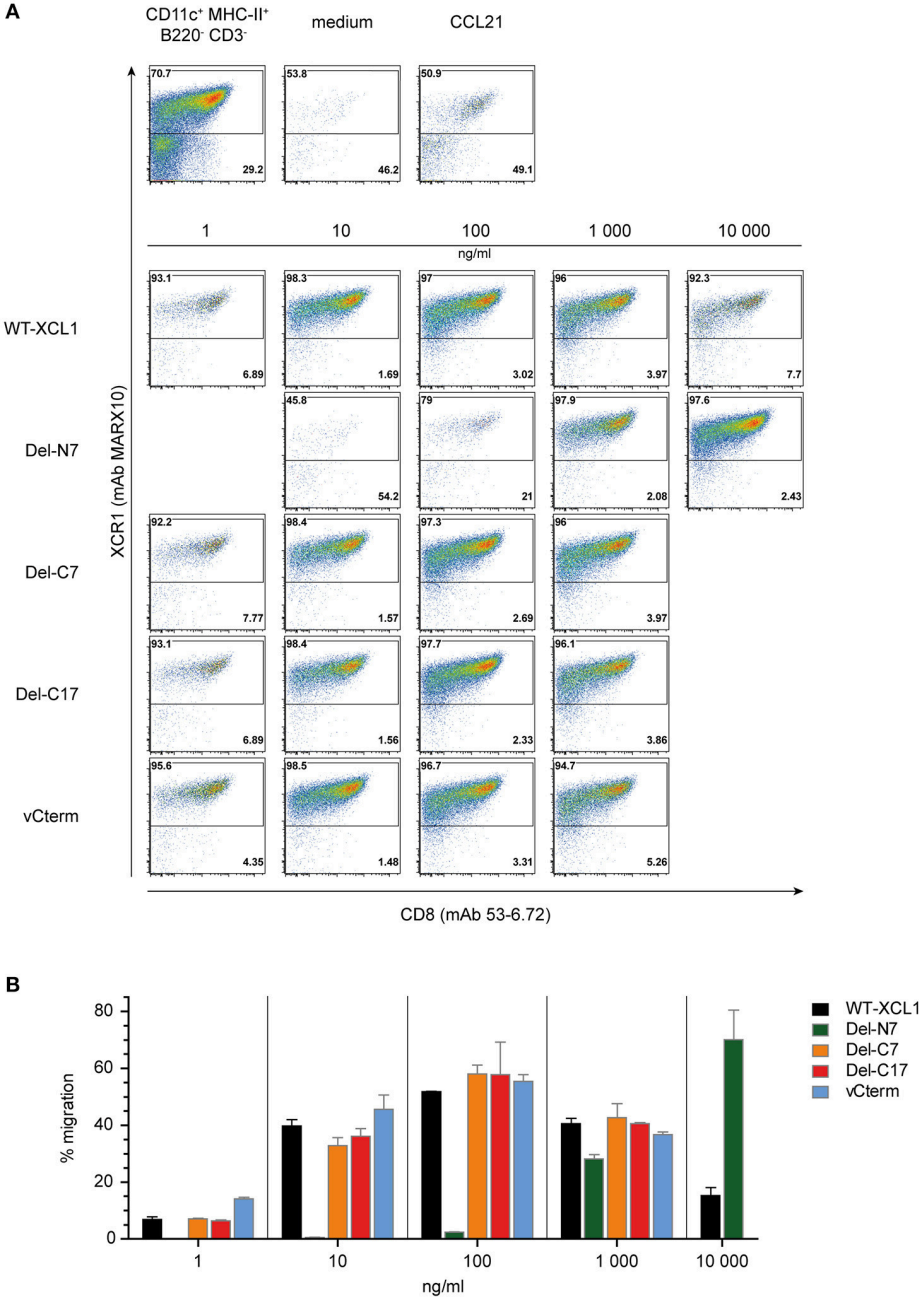
Chemotaxis induced by XCL1-OVA and its variants Del-N7, Del-C7, Del-C17, and vCterm. **(A)** DC enriched from splenocytes by density gradient centrifugation were placed in the upper chamber of a transwell system, the composition of input DC is shown in the leftmost dotplot in the upper pannel. Various concentrations of wt XCL1-OVA and the structural variants were established in the lower chamber and migration of cells was allowed for 2 h. All concentrations are given based on the XCL1-component of the constructs. Thereafter, all cells from the lower chambers were quantitatively analyzed by flow cytometry after staining for XCR1 (mAb MARX10 binds to XCR1 independent of XCL1) and CD8 (mAb 53–6.2). Therefore, the number of events in the dot plots directly represent the number of cells detected. The effect of the negative (medium) and the positive controls (CCL21) is shown in the upper pannels of dot plots. **(B)** Quantitave evaluation of migrated XCR1^+^ DC expressed as percentage of input XCR1^+^ DC of the experiment shown in **(A)**. One experiment representative of 2 independent experiments (each independent experiment was done in duplicate on the same day and the data were combined (mean ± SEM).

In order to determine the functional capacity of the XCL1-OVA structural variants, DC were enriched from splenocytes and tested at various concentrations of the variants for chemotaxis in a transwell system. All of the cells which have migrated into the lower chamber were quantitatively analyzed using flow cytometry; therefore the intensity of the dot-plots truly represents the number of migrated cells. The DC in the input cell population were composed of around 70% of XCR1^+^ DC and 30% XCR1^−^ DC (Figure 3A, leftmost dotplot in upper panel). Virtually no migration into the lower chamber was observed in the medium control, while both XCR1^+^ and XCR1^−^ DC migrated equally well to the chemokine CCL21, which was used as a positive control (Figure 3A, upper panels). Essentially only XCR1^+^ DC migrated in response to the various XCL1-OVA constructs, with virtually no response of T cells, B cells or other XCR1^−^ cells (data not shown). The migration of XCR1^+^ DC to various concentrations of the XCL1-OVA standard (1 ng/ml−10,000 ng/ml) exhibited the bell-shaped curve typical for chemokines, with maximal chemotactic activity at 100 ng/ml (Figures 3A,B). A similar pattern was observed for Del-C7, Del-C17, and also for vCterm (Figures 3A,B). With Del-N7, barely any chemotaxis was observed up to 100 ng/ml and even at 1,000 ng/ml the efficiency did not reach the maximum seen with wt XCL1-OVA. Interestingly, by further increasing the concentration of Del-N7 to 10,000 ng/ml in the lower chamber, Del-N7 was chemotactically active above the levels seen with optimal amounts (100 ng/ml) of wt XCL1-OVA (Figures 3A,B). This was a consistent phenomenon throughout the experiments.

The results obtained in this functional experiment were congruent with the previous binding studies. All XCL1-OVA versions exhibiting good binding also induced effective chemotaxis. Since the Del-C17 variant was similarly active compared to XCL1-OVA, it can be concluded that the C-terminal 17 aa of XCL1 do not participate in the induction of chemotaxis and thus must have other function(s). The positive functional data obtained with Del-N7 XCL1 at very high concentrations indicate that the core domain of XCL1 between aa 8 and 76 contains all necessary structural elements to induce chemotaxis. The first 7 N-terminal aa apparently play a major role in the stabilization of ligand binding to the receptor for induction of chemotaxis. Interestingly, for unknown reason, this N-terminal stretch of XCL1 seems also to limit the signaling at high ligand concentrations.

### Binding of XCL1-OVA and Its Variants Del-N7, Del-C7, Del-C17, and vCterm to Apoptotic and Necrotic Cells

**Figure 4:**
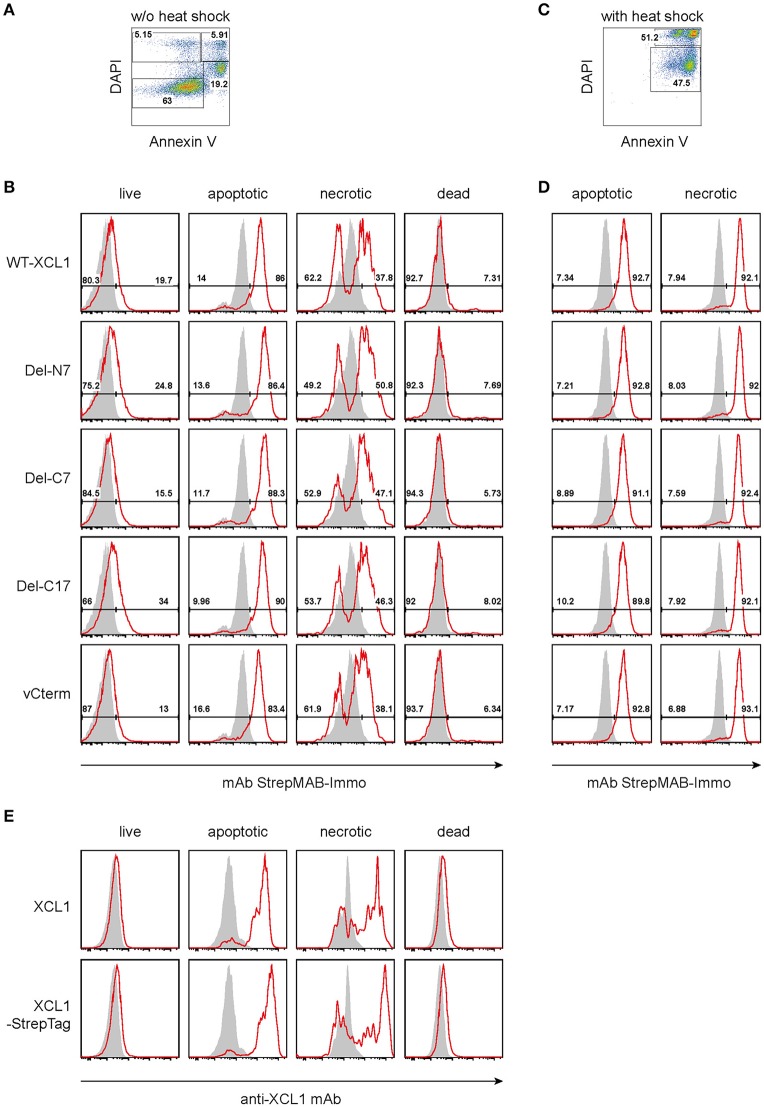
Binding of XCL1-OVA and its variants Del-N7, Del-C7, Del-C17, and vCterm to apoptotic and necrotic cells. P3X63Ag8.653 cells were either **(A,B,E)** cultured at standard conditions without stress, or **(C,D)** subjected to thermal stress (52°C for 15 min, followed by culture overnight). For the last hour of culture, 1 μg of wt XCL1-OVA or one of its variants were added to the culture. For analysis, the cells were washed, and stained with DAPI and AnnexinV to subdivide the cells into “live” (Annexin^−^DAPI^−^), “apoptotic” (AnnexinV^+^DAPI^low^), “necrotic” (Annexin^+^DAPI^+^), and “dead” (Annexin^−^DAPI^+^) cells. **(A) G**ating and **(B)** staining of cells without thermal stress, **(C)** gating and **(D)** staining of cells after thermal stress, using anti-StrepMAB-Immo for signal detection (red histograms); background staining with StrepMAB-Immo without any preincubation is shown in gray histograms. **(E)** P3X63Ag8.653 cells were cultured under identical conditions, without thermal stress. For the last hour of culture, 1 μg of wt XCL1 or XCL1-StrepTag were added to the culture. Washing of cells and gating with DAPI and AnnexinV was as described above. Signal was detected with mAb MTAC-311 specific for murine XCL1 (red histograms), background signals with MTAC-311, without any preincubation, are shown in gray. Concentrations of XLC1-variants are based on the XCL1-component of the respective construct.

As outlined in the introduction, XCL1 is an integral part of the Th1-defense. Given the secretion of XCL1 by activated NK cells, Th1-polarized CD4^+^ T cells, and by activated CD8^+^ T cells, we tested the hypothesis that secreted XCL1 could “mark” stressed cells and thus facilitate their uptake by cross-presenting DC. To test this hypothesis, we examined the binding of wt XCL1-OVA and its structural variants to stressed cells. To this end, P3X63Ag8.653 myeloma cells obtained from standard culture (and thus without stress signals) were double-stained with DAPI and Annexin V and arbitrarily subdivided into populations designated as “live” (Annexin V^−^ DAPI^−^), “apoptotic” (Annexin V^+^ DAPI^low^), “necrotic” (Annexin V^+^ DAPI^+^), and “dead” (Annexin V^−^ DAPI^+^) (Figure 4A). While live and dead cells did not exhibit a strong binding of the various reagents, apoptotic and necrotic cells bound each reagent to a substantial degree, with a rather uniform staining pattern (Figure 4B, background staining with StrepMAB-Immo in gray). When the P3X cells were subjected to thermal stress (52°C for 15 min, followed by overnight culture), again both necrotic and apoptotic cells bound the various reagents in an uniform fashion (Figures 4C,D). To determine which component(s) of the constructs was responsible for the observed binding, additional experiments were performed. Live, apoptotic, necrotic, and dead cells were reacted with XCL1 or with XCL1-StrepTag and any bound reagent detected with a mAb directed to murine XCL1 (Figure 4E). Only apoptotic and necrotic cells gave the characteristic signal pattern, after incubation with either XCL1 or XCL1-StrepTag. Since both reagents gave a staining pattern similar to the other constructs (Figure 4B), any significant binding of StrepTag or OVA to stressed cells could be excluded. Together, these experiments determined that XCL1 was responsible for binding to stressed cells. Furthermore, it could be concluded that the structured core region of XCL1 was responsible for the binding to stressed cells, with no obvious contribution of the free N-terminal or C-terminal regions. Altogether, the data were compatible with the notion that XCL1 marks stressed cells.

### Induction of CD8^+^ T Cell Proliferation and Cytotoxic Capacity After *in vivo* Targeting of Antigen With XCL1-OVA and Its Variants Del-N7, Del-C7, Del-C17, and vCterm

**Figure 5:**
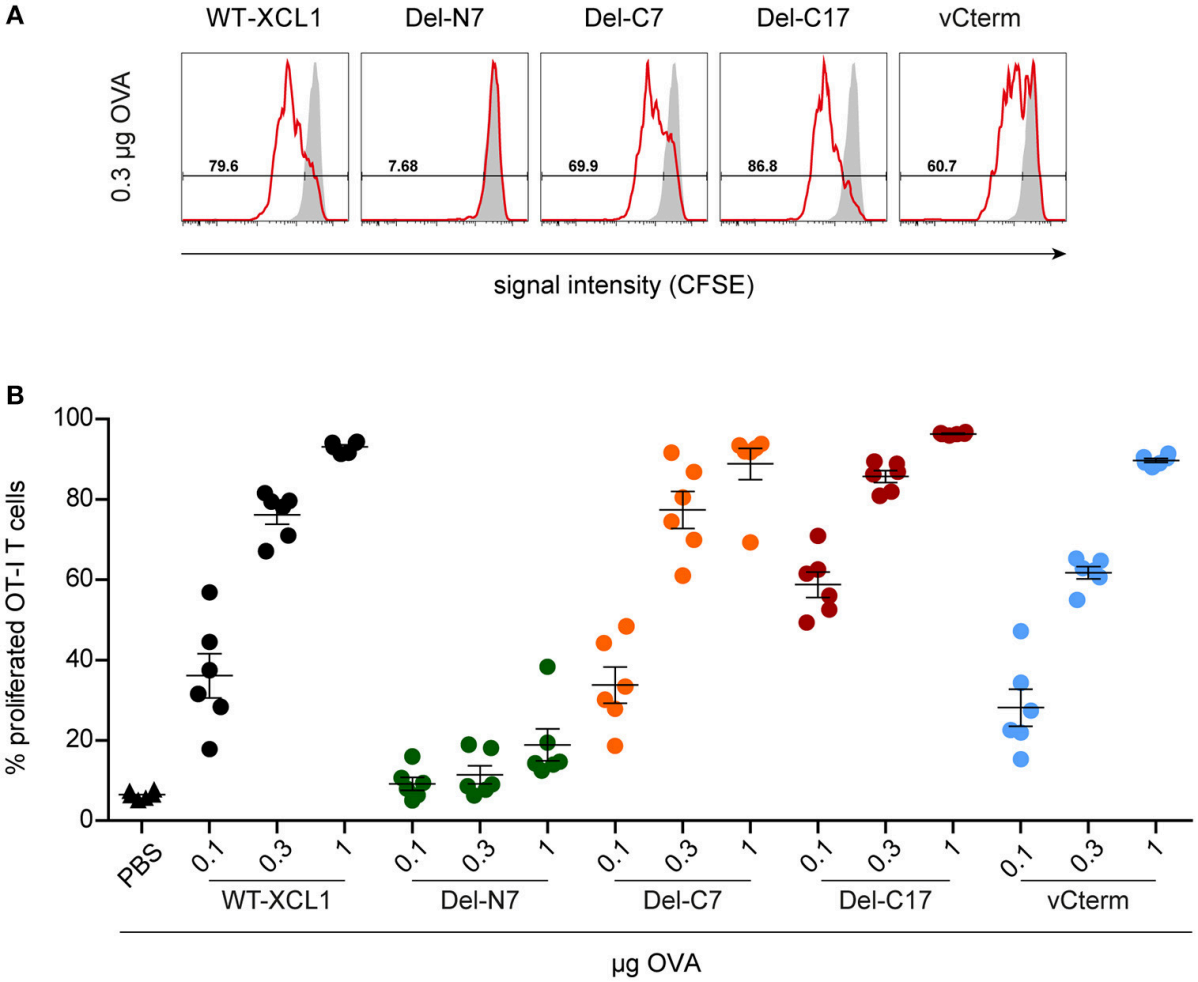
Induction of CD8^+^ T cell proliferation after *in vivo* targeting of antigen using XCL1-OVA and its variants Del-N7, Del-C7, Del-C17, and vCterm. Fluorescently labeled OT-I T cells (1 × 10^6^) were adoptively transferred into syngeneic C57BL/6 mice. One day later the animals were immunized with the indicated amounts (based on the content of OVA) of wt XCL1-OVA and its structural variants (w/o adjuvant). Two days after immunization, the spleens were removed and the percentage of proliferated OT–I T cells was determined using the CSFE dilution assay in flow cytometry. **(A)** Exemplary histograms of the fluorescence signal obtained with 0.3 μg of each protein reagent. **(B)** Percentage of proliferated OT-I T cells after immunization with 0.1, 0.3, and 1 μg of each protein reagent. Combined data from 2 independent experiments are shown (mean ± SEM).

In the next experiments we tested whether the various structural elements of XCL1 influence targeting of antigen *in vivo*. To this end, fluorescently labeled OT–I T cells were adoptively transferred into syngeneic C57BL/6 mice. One day later, mice were immunized i.v. with various amounts (0.1 μg, 0.3 μg, 1 μg) of wt XCL1-OVA, or alternatively with equal amounts of its variants Del-N7, Del-C7, Del-C17, and vCterm without adjuvant; PBS was used as negative control. After 48 h, spleens were removed and the proliferation of the OT–I T cells determined through dilution of the fluorescence signal using flow cytometry. In two independent experiments, Del-C17 induced higher proliferation of the OT–I CD8^+^ T cells at lower dosages, possibly reflecting its higher binding capacity (see Figure 2). vCterm was somewhat less effective, while Del-C7 was comparable to the wt XCL1-OVA standard regarding CD8^+^ T cell proliferation (Figures 5A,B). Del-N7 gave at 1 μg a very subtle proliferation signal (Figure 5B). This experiment demonstrated a comparably effective targeting of XCL1-OVA *in vivo* into DC by all XCL1-OVA variants with comparable binding capacity *in vitro*. This observation excluded a major effect of the C-terminal portion of XCL1 on OVA processing and presentation.

### Induction of Cytotoxic Capacity After *in vivo* Targeting of Antigen With XCL1-OVA and Its Variants Del-N7, Del-C7, Del-C17, and vCterm

**Figure 6:**
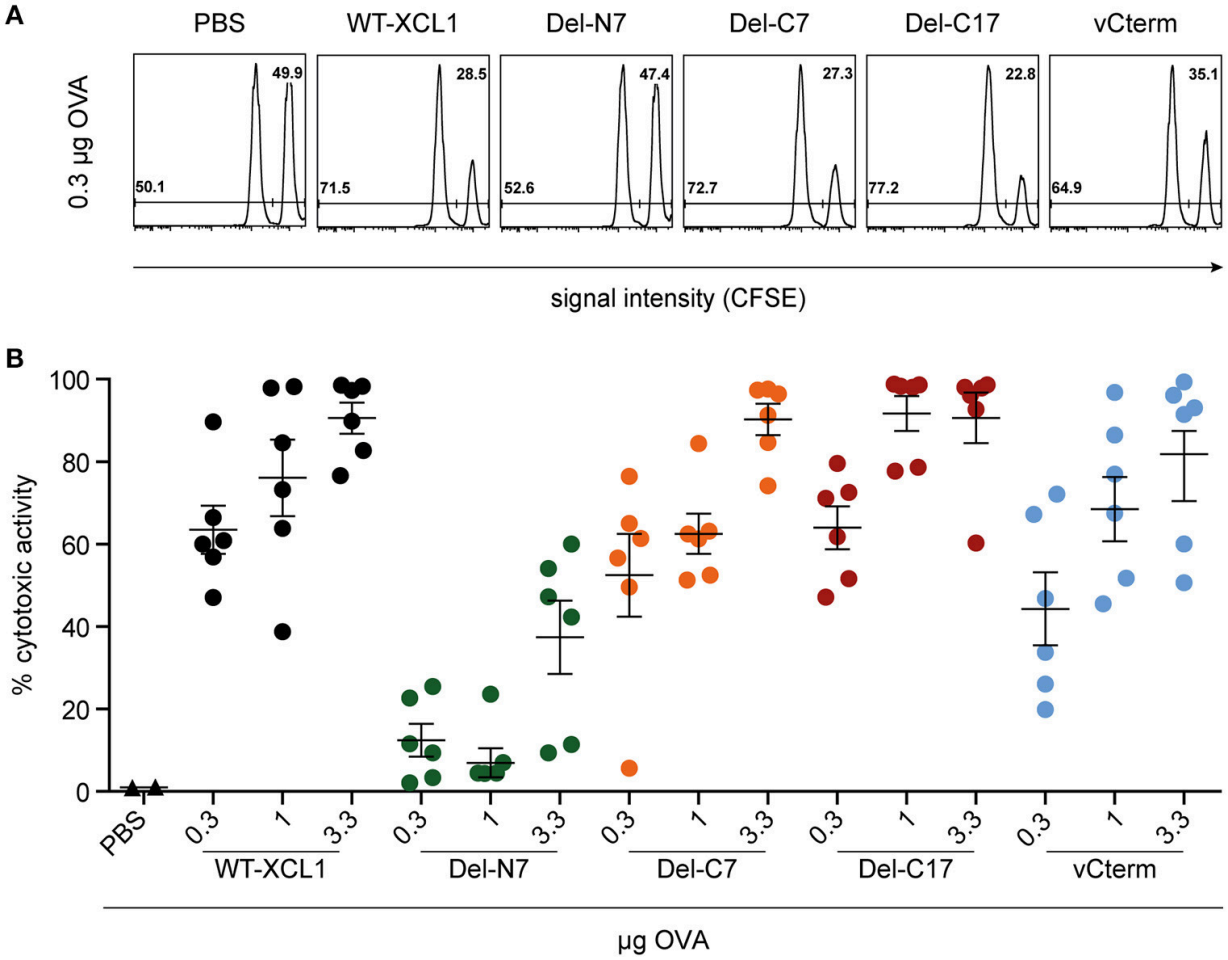
Induction of cytotoxic capacity after *in vivo* targeting of antigen with XCL1-OVA and its variants Del-N7, Del-C7, Del-C17, and vCterm. C57BL/6 mice were immunized with the indicated amounts of wt XCL1-OVA and its variants (based on the content of OVA) together with 3 μg LPS on day 0. On day 6, the animals were injected with CFSE-labeled target cells to quantitate the induced cytotoxicity *in vivo* (for details, see Materials and Methods). **(A)** Exemplary flow cytometry histograms of the data obtained with 0.3 μg of each reagent. **(B)** Percentage of specific lysis obtained with 0.3, 1 and 3.3 μg of each reagent (mean ± SEM).

To further test the *in vivo* functional capacity of T cells induced by the various formats of targeted antigen, C57BL/6 mice were immunized i.v. with titrated amounts of wt XCL1-OVA and its variants, which were injected together with 3 μg LPS. On day 6, an *in vivo* cytotoxicity assay was performed. As can be seen from Figure 6, Del-C7, Del-C17, and vCterm were similarly effective in inducing cytotoxicity as XCL1-OVA. The Del-N7 variant, at 3.3 μg, also induced modest cytotoxic activity. The cytotoxicity results were thus congruent with the proliferation data of OT–I T cells obtained earlier and excluded a major effect of the C-terminal portion of XCL1 on the induction of cytotoxicity *in vivo*.

## Discussion

Structures of many chemokines have been solved by NMR and X-ray crystallography. These studies revealed that despite low sequence homology, chemokines adopt a remarkably conserved tertiary structure consisting of a disordered N-terminus of 6–10 aa, a structured core region (chemokine fold), and a disordered C-terminus of variable length (27, 28). From a great number of structure-function studies a general concept evolved which assumes an initial specific binding of the chemokine fold-domain to the N-terminus of the receptor. In a second step, this initial interaction is stabilized by a subsequent integration of the N-terminal disordered domain of the ligand into the orthosteric pocket of the receptor. Additional studies suggested that this N-terminal domain of the chemokine ligand has signaling capacity. For example, an N-terminal deletion variant of MCP-1_9−76_ bound to its receptor with similar strength compared to the wild-type version MCP-1_1−76_, but had lost all chemotactic activity (29). Based on many experiments of this type, the disordered N-terminal region of chemokines is generally regarded as a key signaling domain (27, 28).

In our work, the N-terminal deletion variant Del-N7 (XCL1_8−93_) reduced the binding of murine XCL1 to its receptor XCR1 approximately 50-fold. This reduction in binding was accompanied by a similar reduction in chemotaxis. The capacity of XCL1 to bind to its receptor was thus directly correlated to its chemotactic action. These data are compatible with a stabilization of the ligand-receptor interaction and thus with the general concept. However, we also made the surprising observation that at very high concentrations (approximately 100-fold of the presumed physiological concentration), this N-terminal deletion variant still exhibited fully preserved chemotactic action. Thus, with murine XCL1 there is no indication of an important signaling element in the N-terminal disordered domain which would be required for chemotaxis, as suggested by the general concept. Interestingly, at these supra-physiological concentrations (10,000 ng/ml) the N-terminal deletion variant consistently induced higher chemotaxis, when compared to the wild-type XCL1 at its optimal concentration (100 ng/ml). This observation indicates that the chemokine fold of XCL1 contains all necessary structures to induce chemotaxis. Finally, wild-type XCL1 at the same supra-physiological concentrations (10,000 ng/ml), exhibited largely reduced chemotaxis compared to its optimum at 100 ng/ml (as is typical for chemokines). This observation suggest that the disordered N-terminal region of XCL1 in some ways limits the functional activity of XCL1 at high concentrations.

When we analyzed the functional contribution of the C-terminal portion of murine XCL1, the deletion variants Del-C7 (XCL1_1−86_) and Del-C17 (XCL1_1−76_) did not show any functional effects on receptor binding or chemotaxis. Thus, we can exclude a major contribution of this region to the chemotactic function of murine XCL1. This conclusion clearly differs from the findings of a study utilizing human XCL1_1−72_ (30), where a complete loss of calcium activity was observed. However, this particular C-terminal deletion variant was 4 aa shorter than Del-C17 (XCL1_1−76_) which was used in the present study. Our results are fully compatible with data on a series of C-terminally truncated variants of human XLC1 (1–68, 1–72, 1–78, and 1–85), which all elicited normal calcium signals in XCR1-transfected HEK-293 cells (31).

Regarding the structure-function relationship of murine XCL1, our data can be summarized as follows. The core domain of XCL1 contains all necessary structural elements to specifically bind to XCR1 and to elicit chemotaxis. This observation differs from the general concept for chemokines, which assumes a critical signal contribution of the N-terminal domain for chemotaxis (27, 28). The first 7 aa of the N-terminal domain stabilize the binding of XCL1 to its receptor and thus certainly optimize chemotaxis. At the same time, and apparently paradoxically, the first 7 aa appear to limit the chemotactic effect of supra-physiological concentrations of murine XCL1, suggesting some type of negative regulatory role of the N-terminus at high XCL1 concentrations. A contribution of the disordered C-terminus of XCL1 to chemotaxis can clearly be ruled out.

These conclusions were reached with binding and chemotaxis assays using primary cells expressing the native XCR1 receptor. This is in contrast to the very few studies on the structure-function relationship of XCL1, which were conducted with XCR1-transfectants and mainly based on calcium mobilization studies.

Since XCL1 is secreted by activated NK cells, we pursued the hypothesis that in addition to its chemotactic function, XCL1 could “decorate” stressed cells recognized by NK cells. In such a scenario bound XCL1 could mark these stressed cells for phagocytosis by DC specialized for uptake of such cells (16). Using a murine myeloma line as an *in vitro* model system and also employing heat-shock experiments, we consistently observed binding of XCL1 to cells characterized as “necrotic” or “apoptotic,” based on the use of Annexin V and DAPI. This binding was clearly mediated by the chemokine fold of XCL1, with no apparent contribution of the disordered N-terminal or C-terminal regions. It is unclear at present, to which structural elements present on apoptotic and necrotic cells XCL1 binds. Therefore, it remains to be determined whether this binding is specific or mediated by structural elements common to many chemokines, e.g., domains capable to mediate binding to glycosaminoglycans (present in the core domain of chemokines, also with XCL1). Preliminary studies with primary cells gave similar results as with the myeloma line, but turned out to be less reproducible, and therefore more work is needed to reach firm conclusions here. In particular, *in vivo* work will be required to generate essential data in order to support or reject the “decoration” hypothesis.

We are interested to use XCL1 as a carrier to transport proteins or peptides into cross-presenting DC. Therefore, all experiments were performed with XCL1 variants which were C-terminally fused to full-length OVA. We wanted to determine whether the various domains of XCL1 exert any influence on the targeting of the model protein to XCR1^+^ DC *in vivo*. As independent and very sensitive readouts for CD8^+^ T cell activation we used both induction of proliferation (response by adoptively transferred OT-I T cells) as well as induction of cytotoxic activity (by endogenous CD8^+^ T cells). Since CD8^+^ T cells *in vivo* become activated through cross-presentation of the soluble antigen OVA (17–19), we assume that these readouts measure cross-presentation of OVA-derived peptide SIINFEKL by XCR1^+^ DC *in vivo*. They thus reflect the combined effects of antigen uptake, efficiency of antigen processing, and antigen presentation on the cell surface of the DC. Previous experiments which demonstrated that targeting of OVA using either XCL1 or an antibody directed to murine XCR1 drastically reduces the amount of antigen necessary to elicit CD8^+^ T cell responses *in vivo* (20) support this assumption.

Several conclusions can be reached from our experiments regarding the use of XCL1 as antigen carrier. First, an intact N-terminus is required to efficiently target any peptide/protein cargo to XCR1^+^ DC. Second, attachment of a relatively large protein cargo of around 40 kDa to the C-terminus does not sterically inhibit binding of XCL1 to its receptor. Third, attachment of protein cargo does not influence chemotaxis of XCR1^+^ DC (chemotaxis was identical when compared with native XCL1 without OVA, data not shown). Since a chemotactic signal usually induces internalization of the chemokine receptor (27, 28), we assume that fusion of protein cargo to XCL1 does not influence the uptake of the protein into XCR1^+^ DC. Fourth, the disordered C-terminus can be eliminated from XCL1, if necessary, when using protein cargo without any obvious deficits in antigen uptake and presentation. The last conclusion is supported by exchanging the natural C-terminus of XCL1 with the C-terminal domain of murid herpesvirus 8-encoded XCL1, which also did not show any changes in antigen presentation.

The conclusions on the capacity of XCL1 as an antigen carrier were reached with the model antigen OVA. Since this particular antigen is ideal in that it is highly soluble and shows little interaction with other proteins, there may be some limitations to the conclusions reached. Other proteins prone to binding to other structures in the body may not as efficiently be transported to XCR1^+^ DC as OVA. Other cargo proteins may also interact with XCL1 in an intra- or intermolecular fashion. In spite of these potential limitations, our data clearly demonstrate the usefulness of XCL1 as a carrier to directly target large proteins or peptides to XCR1^+^ cross-presenting DC. Such an antigen carrier system appears attractive for induction of antigen-specific cytotoxicity in anti-tumor therapeutic vaccines.

## Ethics Statement

This study was carried out in accordance with the recommendations of name of guidelines, name of LAGS Berlin. The protocol was approved by the LAGS Berlin.

## Author Contributions

ALK performed all experiments and wrote parts of the manuscript. EH, NR, MB, and SG assisted in some of the biological experiments. HWM assisted in the molecular biology experiments. SV contributed information on the viral XCL1. CF and RAK designed the experiments. RAK wrote the manuscript.

### Conflict of Interest Statement

RAK is inventor on a patent held by the Robert Koch-Institute on targeting of antigens via XCR1. The remaining authors declare that the research was conducted in the absence of any commercial or financial relationships that could be construed as a potential conflict of interest.

## Abbreviations

DC: dendritic cells
OVA: ovalbumin
MFI: mean fluorescence intensity.

## References

[1] Kelner GS Kennedy J Bacon KB Kleyensteuber S Largaespada DA Jenkins NA. Lymphotactin: a cytokine that represents a new class of chemokine. Science (1994) 266:1395–9. 10.1126/science.7973732 7973732

[2] Müller S Dorner B Korthäuer U Mages HW D'Apuzzo M Senger G. Cloning of ATAC, an activation-induced, chemokine-related molecule exclusively expressed in CD8^+^ T lymphocytes. Eur J Immunol. (1995) 25:1744–8. 10.1002/eji.1830250638 7615002

[3] Yoshida T Imai T Kakizaki M Nishimura M Yoshie O. Molecular cloning of a novel C or γ type chemokine, SCM-1. FEBS Lett. (1995) 360:155–9. 10.1016/0014-5793(95)00093-O 7875320

[4] Geyer H Hartung E Mages HW Weise C Belužić R Vugrek O. Cytomegalovirus expresses the chemokine homologue vXCL1 capable of attracting XCR1^+^ CD4^−^ dendritic cells. J. Virol. (2014) 88:292–302. 10.1128/JVI.02330-13 24155383 PMC3911744

[5] Dorner BG Scheffold A Rolph MS Hüser MB Kaufmann SH Radbruch A. MIP-1α, MIP-1β, RANTES, and ATAC/lymphotactin function together with IFN-γ as type 1 cytokines. Proc Natl Acad Sci USA. (2002) 99:6181–6. 10.1073/pnas.092141999 11972057 PMC122923

[6] Kroczek RA Henn V. The Role of XCR1 and its ligand XCL1 in antigen cross-presentation by murine and human dendritic cells. Front Immunol. (2012) 3:14. 10.3389/fimmu.2012.00014 22566900 PMC3342032

[7] Yoshida T Izawa D Nakayama T Nakahara K Kakizaki M Imai T. Molecular cloning of mXCR1, the murine SCM-1/ lymphotactin receptor. FEBS Lett. (1999) 458:37–40. 10518929 10.1016/s0014-5793(99)01114-x

[8] Shan L Qiao X Oldham E Catron D Kaminski H Lundell D. Identification of viral macrophage inflammatory protein (vMIP)-II as a ligand for GPR5/XCR1. Biochem Biophys Res Commun. (2000) 268:938–41. 10.1006/bbrc.2000.2235 10679309

[9] Dorner BG Dorner MB Zhou X Opitz C Mora A Güttler S. Selective expression of the chemokine receptor XCR1 on cross-presenting dendritic cells determines cooperation with CD8^+^ T cells. Immunity (2009) 31:823–33. 10.1016/j.immuni.2009.08.027 19913446

[10] Bachem A Güttler S Hartung E Ebstein F Schaefer M Tannert A. Superior antigen cross-presentation and XCR1 expression define human CD11c^+^CD141^+^ cells as homologues of mouse CD8^+^ dendritic cells. J Exp Med. (2010) 207:1273–81. 10.1084/jem.20100348 20479115 PMC2882837

[11] Crozat K Guiton R Contreras V Feuillet V Dutertre CA Ventre E. The XC chemokine receptor 1 is a conserved selective marker of mammalian cells homologous to mouse CD8α^+^ dendritic cells. J Exp Med. (2010) 207:1283–92. 10.1084/jem.20100223 20479118 PMC2882835

[12] Crozat K Tamoutounour S Vu Manh TP Fossum E Luche H Ardouin L. Cutting edge: expression of XCR1 defines mouse lymphoid-tissue resident and migratory dendritic cells of the CD8α^+^ type. J Immunol. (2011) 187:4411–5. 10.4049/jimmunol.1101717 21948982

[13] Bachem A Hartung E Güttler S Mora A Zhou X Hegemann A. Expression of XCR1 characterizes the Batf3-dependent lineage of dendritic cells capable of antigen cross-presentation. Front Immunol. (2012) 3:214. 10.3389/fimmu.2012.00214 22826713 PMC3399095

[14] Iyoda T Shimoyama S Liu K Omatsu Y Akiyama Y Maeda Y. The CD8^+^ dendritic cell subset selectively endocytoses dying cells in culture and in vivo. J Exp Med. (2002) 195:1289–302. 10.1084/jem.20020161 12021309 PMC2193756

[15] Schulz O Reis e Sousa C. Cross-presentation of cell-associated antigens by CD8α^+^ dendritic cells is attributable to their ability to internalize dead cells. Immunology (2002) 107:183–9. 10.1046/j.1365-2567.2002.01513.x 12383197 PMC1782783

[16] Shortman K Heath WR. The CD8^+^ dendritic cell subset. Immunol Rev. (2010) 234:18–31. 10.1111/j.0105-2896.2009.00870.x 20193009

[17] Kurts C Kosaka H Carbone FR Miller JF Heath WR. Class I-restricted cross-presentation of exogenous self-antigens leads to deletion of autoreactive CD8^+^ T cells. J Exp Med. (1997) 186:239–45. 10.1084/jem.186.2.239 9221753 PMC2198972

[18] den Haan JM Lehar SM Bevan MJ CD8^+^ but not CD8^−^ dendritic cells cross-prime cytotoxic T cells in vivo. J Exp Med. (2000) 192:1685–96. 10.1084/jem.192.12.1685 11120766 PMC2213493

[19] Pooley JL Heath WR Shortman K. Cutting edge: intravenous soluble antigen is presented to CD4 T cells by CD8^−^ dendritic cells, but cross-presented to CD8 T cells by CD8^+^ dendritic cells. J Immunol. (2001) 166:5327–30. 10.4049/jimmunol.166.9.5327 11313367

[20] Hartung E Becker M Bachem A Reeg N Jäkel A Hutloff A Induction of potent CD8 T cell cytotoxicity by specific targeting of antigen to cross-presenting dendritic cells in vivo via murine or human XCR1. J Immunol. (2015) 194:1069–79. 10.4049/jimmunol.1401903 25520399

[21] Kuloglu ES McCaslin DR Kitabwalla M Pauza CD Markley JL Volkman BF. Monomeric solution structure of the prototypical 'C' chemokine lymphotactin. Biochemistry (2001) 40:12486–96. 10.1021/bi011106p 11601972 PMC3826542

[22] Kuloglu ES McCaslin DR Markley JL Volkman BF. Structural rearrangement of human lymphotactin, a C chemokine, under physiological solution conditions. J Biol Chem. (2002) 277:17863–70. 10.1074/jbc.M200402200 11889129 PMC4451178

[23] Wallny H Production of MHC class II molecules in drosophila melanogaster Schneider cells. In: Lefkovits editor. Immunological Methods Manual Volume 1, San Diego, CA: Academic Press (1997), 51–59.

[24] Schneider I. Cell lines derived from late embryonic stages of Drosophila melanogaster. J Embryol Exp Morphol. (1972) 27:353–65. 4625067

[25] Mach N Gillessen S Wilson SB Sheehan C Mihm M Dranoff G Differences in dendritic cells stimulated in vivo by tumors engineered to secrete granulocyte-macrophage colony-stimulating factor or Flt3-ligand. Cancer Res. (2000) 60:3239–46. 10866317

[26] Löhning M Hutloff A Kallinich T Mages HW Bonhagen K Radbruch A. Expression of ICOS in vivo defines CD4^+^ effector T cells with high inflammatory potential and a strong bias for secretion of interleukin 10. J Exp Med. (2003) 197:181–93. 10.1084/jem.20020632 12538658 PMC2193816

[27] Allen SJ Crown SE Handel TM. Chemokine: receptor structure, interactions, and antagonism. Ann Rev Immunol. (2007) 25:787–820. 10.1146/annurev.immunol.24.021605.090529 17291188

[28] Bachelerie F Ben-Baruch A Burkhardt AM Combadiere C Farber JM Graham GJ. International union of basic and clinical pharmacology. [corrected] LXXXIX Update on the extended family of chemokine receptors and introducing a new nomenclature for atypical chemokine receptors. Pharmacol Rev. (2014) 66:1–79. 10.1124/pr.113.007724 24218476 PMC3880466

[29] Gong JH Clark-Lewis I. Antagonists of monocyte chemoattractant protein 1 identified by modification of functionally critical NH2-terminal residues. J Exp Med. (1995) 181:631–40. 10.1084/jem.181.2.631 7836918 PMC2191888

[30] Marcaurelle LA Mizoue LS Wilken J Oldham L Kent SB Handel TM. Chemical synthesis of lymphotactin: a glycosylated chemokine with a C-terminal mucin-like domain. Chemistry (2001) 7:1129–32. 10.1002/1521-3765(20010302)7:53.0.CO;2-W 11303872

[31] Tuinstra RL Peterson FC Elgin ES Pelzek AJ Volkman BF. An engineered second disulfide bond restricts lymphotactin/XCL1 to a chemokine-like conformation with XCR1 agonist activity. Biochemistry (2007) 46:2564–73. 10.1021/bi602365d 17302442 PMC2734904

